# The Effectiveness of Functional Septorhinoplasty in Improving COVID-19-related Olfactory Dysfunction

**DOI:** 10.1055/a-2535-0153

**Published:** 2025-02-24

**Authors:** Alfonso Luca Pendolino, Bruno Scarpa, Peter J. Andrews

**Affiliations:** 1 Department of ENT, Royal National ENT & Eastman Dental Hospitals, London, United Kingdom; 2 Ear Institute, UCL, London, United Kingdom; 3 Department of Statistical Sciences and Department of Mathematics Tullio Levi-Civita, University of Padova, Padova, Italy

**Keywords:** olfaction, olfactory dysfunction, post-infectious olfactory dysfunction, COVID-19, septorhinoplasty

## Abstract

Long-Term COVID-19-related olfactory dysfunction (C19OD) remains a significant challenge with no established treatment providing meaningful improvement. This study aimed to assess the efficacy of functional septorhinoplasty (fSRP) in improving olfactory dysfunction in patients with persistent C19OD compared to a control group undergoing olfactory training (OT). In this prospective study patients with persistent C19OD undergoing fSRP were enrolled while those declining surgery continued with OT as the control group. Patients were followed for six months with olfactory function assessed using Sniffin' Sticks (S'S) and nasal airflow evaluated through peak nasal inspiratory flow (PNIF) and acoustic rhinometry (AR). Among the participants 12 underwent fSRP while 13 were in the control group. Significant improvements (p < 0.05) in all S'S scores were observed in the fSRP group but not in the control group. TDI scores improved above the minimal clinically important difference only in the fSRP group. Strong correlations were found between olfactory scores and nasal measurements. Comparison of olfactory threshold gains between groups revealed a statistically significant benefit in the fSRP group. These findings suggest that fSRP can significantly improve persistent C19OD providing a notable olfactory threshold gain compared to OT.

The recent COVID-19 pandemic has left millions of people with a profound loss of chemosensation due to the high prevalence of olfactory dysfunction (OD) linked to severe acute respiratory syndrome coronavirus 2 (SARS-CoV-2) infection. 1 2 3 4 Although a high recovery rate has been observed during the first months, 5 up to 13% of subjects can show persistent COVID-19-related OD (C19OD) at 3 years, 6 with severe impact on quality of life (QoL). 3 4

The olfactory pathophysiology of C19OD is multifactorial. Traditionally, causes of OD have been classidied according to the anatomical location of the presumed pathology/lesion and divided as conductive, sensorineural, and central, or a combination of these. SARS-CoV-2 typically damages the olfactory epithelium (OE), thus creating a sensorineural loss of smell, and to a lesser extent affects the central primary and secondary olfactory cortices. 7 8 Although C19OD represents a reversible sensorineural olfactory loss in the majority of cases, 6 the question remains as to why this is not the case for those developing a persistent loss of sense of smell.

Normal nasal airflow through the olfactory cleft is one of the conditions necessary for an intact olfactory sense. 9 Current evidence shows that a moderate to severe deviated nasal septum (DNS) results in decreased olfactory function on the obstructed nasal side (lateralized olfaction) and olfaction is normalized following septoplasty. 10 OD caused by a structural obstruction is mainly caused by a conductive loss secondary to a reduction in the access of odorants to the OE and once rectified the sense of smell returns to normal. 11 12 13 14 15 The relationship between nasal airway improvement following septal surgery and improved olfaction has been consistently demonstrated. 9 15 16 17 18 19 20 21 22 23 24 25 26 27

Functional septorhinoplasty (fSRP), as well as correcting a DNS, can also increase internal/external nasal valve (INV/ENV) function, which is crucial in regulating airflow to the olfactory region. 28 29 With additional INV and ENV augmentation, there is growing evidence suggesting that fSRP can improve olfaction to a greater extent than septoplasty alone and implying that other mechanisms, in addition to the conductive component, are involved in the smell improvement. 10 17 25 26 30 In this regard, Whitcroft et al 10 demonstrated that fSRP can improve olfaction in patients with a combination of conductive and sensorineural olfactory loss. The authors hypothesized that the observed olfactory improvement was achieved by an improved OE function caused by an increased nasal airflow to the olfactory niche. 10

In the post-COVID-19 era, the unmet need is to find a treatment which could achieve a meaningful olfactory increase for patients with long-term (>2 years) C19OD. This noticeable—perceptible for the patient—improvement in the smell function is usually defined as an increase in the smell scores above the minimal clinically important difference (MCID).

Currently only few studies have explored treatments for persistent C19OD lasting longer than 1 year but MCID in olfactory gain has never been achieved. 31 We conducted a pilot study to evaluate olfactory changes in patients with persistent C19OD undergoing fSRP and compared these to a control group of C19OD patients on OT.

## Materials and Methods

Participants were recruited from patients seen in the long-COVID smell clinic at the Royal National ENT hospital (University College London Hospitals, London, United Kingdom) between October 2022 and May 2023. Inclusion and exclusion criteria are reported in Table 1 . This study was approved by the Hospital Research Ethic Committee (ref. 14/SC/1180) and was conducted in accordance with the 1996 Declaration of Helsinki. All participants provided full informed written consent prior to participation.

**Table 1:** Study inclusion and exclusion criteria

Inclusion	Exclusion
Age ≥18	Presence of other causes leading/contributing to OD (also confirmed by MRI of the head/sinuses) ^a^
Etiology of OD following a polymerase chain reaction—confirmed diagnosis of SARS-CoV-2 infection	History of PIOD prior to COVID-19
OD confirmed at Sniffin' Sticks and longer than 18 months	Prior nasal/sinonasal/skull base surgery
OD failing to improve on conservative treatments, including OT and oral/topical corticosteroids	Bleeding disorders
Aesthetically unacceptable nasal deformity or reduced nasal airflow caused by a confirmed DNS and/or INV/ENV dysfunction	Blood thinners assumption

Abbreviations: DNS, deviated nasal septum; ENV, external nasal valve; INV, internal nasal valve; MRI, magnetic resonance imaging; OD, olfactory dysfunction; OT, olfactory training; PIOD, post-infectious olfactory dysfunction.

Note: ^a^ These include: congenital olfactory loss, post-traumatic olfactory dysfunction, chronic rhinosinusitis, neoplasms, previous chemotherapy or radiotherapy to the head and neck, neurodegenerative diseases.

Subjects satisfying eligibility criteria were offered fSRP. Those refusing it but willing to take part in the study were asked to continue with OT for the entire study period and formed the control arm. Subjects in the treatment group were assessed at baseline (T _0_ ), 3 months (T _1_ ), and 6 months (T _2_ ) from fSRP. Those in the control group, instead, were assessed at T _0_ and T _2_ only. During the follow-up period, participants were asked to not start any additional treatment potentially influencing olfaction. Compliance with OT in the control group was assessed at T _2_ . fSRP was performed using a standardized external approach involving septoplasty with nasal bone realignment to increase airway symmetry, and INV and ENV augmentation using autologous spreader grafts and columellar strut respectively. All operations were performed by the same team (PJA/ALP) following the same surgical technique.

Sense of smell was evaluated using S'S extended set (Burghart, Medisense) to obtain the odor threshold (T), discrimination (D), and identification (I) scores. 32 Normosmia was attributed where TDI score was ≥30.75, hyposmia where TDI was >16, but <30.75, and functional anosmia if TDI was ≤16. 32 The MCID was defined as a clinically significant improvement corresponding to 5.5 points increase in TDI (our primary outcome), 2.5 points for odour threshold, and 3 points for both odour discrimination and identification. 33 Bilateral and unilateral peak nasal inspiratory flow (PNIF) measures were performed to assess nasal airflow while acoustic rhinometry (AR) was used to obtain unilateral minimal cross-sectional area (MCA) and nasal volume (NV). 34 35 36 QoL was assessed using the 36-Item Short Form Health Survey (SF-36). Self-assessment of olfaction was performed using a visual analogue scale for smell (sVAS—0 represents “sense of smell absent” and 10 “sense of smell not affected”) 2 whereas sinonasal symptoms were evaluated using the 22-item Sino-Nasal Outcome Test (SNOT-22). 37 The Nasal Obstruction Symptom Evaluation (NOSE) scale was used to subjectively assess nasal obstruction. 38 Qualitative olfactory dysfunction (i.e., parosmia/phantosmia) was investigated by asking the participants if the symptom was present or not at the moment of the examination.

### Statistical Analysis

Quantitative variables were summarized using median and interquartile range whereas qualitative variables were described with frequency and percentage. Comparisons of measurements between baseline and follow-ups were performed using the Mann-Whitney test for quantitative variables and the proportion test for dichotomic variables. Pearson correlation index was used to measure associations between quantitative variables. *p* -values were calculated for all tests, and 5% was considered as the critical level of significance. Sample size was determined using a power analysis of independent Mann-Whitney test (two-sided) assuming a difference between means at the end of the study of 5.5 TDI points (MCID) 33 and an equal standard deviation in the two groups of 4 TDI points. Based on that, a minimum of 10 patients in each group were required to reach a power of 81%, with an alpha error of 0.05.

## Results

This study assessed 104 subjects for eligibility. A total of 25 participants were selected with 12 forming the treatment group and 13 entering the control group. The 6-month follow-up period was completed by 9 patients in the treatment group and 10 in the control arm (6-month drop-out rate of 25.0 and 23.1%, respectively). No complications were recorded following fSRP. Demographics and baseline characteristics for the participants, and comparison between groups, are reported in Table 2 .

**Table 2:** General characteristics of the treatment and control groups at baseline

	Treatment group *n* = 12	Control group *n* = 13	*p* -value
Age, median (P25–P75), yr	40.0 (31.5–44.0)	49.0 (30.0–54.0)	0.66
Sex, No. (%)			0.77
Female	9 (75.0%)	8 (61.5%)	
Male	3 (25.0%)	5 (38.5%)	
Length of OD, a median (P25–P75), yr	2.3 (2.0–2.5)	2.4 (1.9–2.8)	0.53
Parosmia, No. (%)	10 (83.3%)	10 (76.9%)	1
Phantosmia, No. (%)	4 (33.3%)	2 (15.4%)	0.56
Smoking, No. (%)			0.50
Ex-smoker	1 (100%)	0 (0.0%)	
Yes	0 (0.0%)	2 (100%)	
No	0 (0.0%)	0 (0.0%)	
Comorbidity, No. (%)			0.33
None	8 (66.7%)	9 (69.2%)	
Yes	4 (33.3%)	4 (30.8%)	
Hypothyroidism	1 (25.0%)	1 (25.0%)	
Asthma	1 (25.0%)	1 (25.0%)	
Others	4 (100%)	3 (75.0%)	
Allergic rhinitis, No. (%)	2 (16.7%)	0 (0.0%)	0.94
Chronic rhinosinusitis, No. (%)	0 (0.0%)	0 (0.0%)	1
Family history Alzheimer/Parkinson, No. (%)	3 (25.0%)	1 (7.7%)	1
History of PIOD, No. (%)	2 (16.6%)	3 (23.1%)	1
History of previous nasal operations, No. (%)	0 (0.0%)	0 (0.0%)	1
History of head trauma, No. (%)	1 (8.3%)	0 (0.0%)	0.97

Abbreviations: OD, olfactory dysfunction; PIOD, post-infectious olfactory dysfunction.

Notes: Statistical difference between groups is also shown. Levels of significance: * *p* ≤ 0.05.

a Length of OD is calculated as number of days from the infection date to the day of enrolment.

### Olfactory Scores, Nasal Measurements, and Patient-Reported Outcome Measures (PROMs) at Baseline

Apart from the median discrimination scores, all S'S subtest scores at baseline were below normative values when compared to those of an adult population of similar age group. 32 Similarly, baseline median bilateral and unilateral PNIF as well as AR parameters were below the reference values for an adult population of similar age group. 35 39 40 Lower SF-36 scores were found for the health domains role limitations due to physical health, energy/fatigue, emotional well-being, social functioning, and general health when compared to normative values for the UK population. 41 Reduced scores were observed for sVAS while raised scores were found for the SNOT-22 42 and NOSE. 43 No statistically significant differences were noted in the olfactory scores, nasal measurements, and PROMs at baseline between the two groups ( Tables 3 and 4 ).

**Table 3:** Olfactory and nasal measurements, and patient-reported outcome measures (PROMs) at baseline, 3 months, and 6 months following functional septorhinoplasty for the treatment group and at baseline and at 6 months for the control group

	*Treatment group*			*Control group*	
	Baseline (T _0_ ) *n* = 12	3 months (T _1_ ) *n* = 10	6 months (T _2_ ) *n* = 9	Baseline (T _0_ ) *n* = 13	6 months (T _2_ ) *n* = 10
**Sniffin' Sticks**					
TDI, median (P25–P75)	22.3 (20.0–24.8)	26.8 (20.0–24.8)	30.3 (24.5–30.8)	22.0 (18.0–25.0)	21.9 (21.1–31.2)
Threshold, median (P25–P75)	1.8 (1.0–3.8)	4.6 (1.7–7.0)	5.8 (4.0–7.3)	4.0 (2.3–4.5)	4.4 (2.2–5.4)
Discrimination, median (P25–P75)	10.0 (10.0–11.3)	11.5 (10.0–12.0)	12.0 (11.0–13.0)	9.0 (8.0–10.0)	10.5 (8.0–13.0)
Identification, median (P25–P75)	9.0 (8.0–11.3)	10.5 (9.0–12.8)	12.0 (10.0–13.0)	9.0 (7.0–10.0)	10.0 (8.3–10.8)
Normosmics, *n* (%)	0 (0.0%)	1 (10.0%)	4 (44.4%)	0 (0.0%)	3 (30.0%)
Hyposmics, *n* (%)	11 (91.7%)	9 (90.0%)	5 (55.6%)	12 (92.3%)	6 (60.0%)
Anosmics, *n* (%)	1 (8.3%)	0 (23.5%)	0 (0.0%)	1 (7.7%)	1 (10.0%)
**Nasal measurements**					
PNIF, median (P25–P75), L/min					
Bilateral PNIF	115.0 (87.5–137.5)	137.5 (130.0–157.5)	160.0 (125.0–190.0)	135.0 (110.0–162.5)	
Right PNIF	62.5 (50.0–82.5)	82.5 (66.3–130.0)	110.0 (70.0–120.0)	82.5 (66.3–98.8)	
Left PNIF	60.0 (48.8–77.5)	90.0 (81.3–107.5)	65.0 (50.0–100.0)	100.0 (68.8–116.3)	
Acoustic rhinometry, median (P25–P75)					−
Right MCA1, cm ^2^	0.5 (0.4–0.7)	0.5 (0.4–0.7)	0.6 (0.4–0.7)	0.6 (0.4–0.9)	
Right nasal volume (0–5), cm ^3^	5.7 (5.2–7.3)	8.1 (5.9–10.9)	7.9 (5.8–9.4)	6.9 (6.2–13.1)	
Left MCA1, cm ^2^	0.7 (0.4–0.8)	0.7 (0.5–0.8)	0.6 (0.5–0.8)	0.8 (0.6–1.1)	
Left nasal volume (0–5), cm ^3^	6.6 (5.7–7.4)	9.5 (7.6–10.7)	8.0 (6.0–9.1)	9.0 (6.1–13.1)	
**PROMs**					−
SF-36, median (P25–P75), %					
Physical functioning	95.0 (83.8–100)	100 (81.3–100)	95.0 (90.0–100)	100 (87.5–100)	100 (91.3–100)
Role limitations due to physical health	62.5 (25.0–100)	75.0 (31.3–100)	75.0 (50.0–100)	100 (75.0–100)	100 (100–100)
Role limitations due to emotional problems	100 (33.3–100)	100 (66.7–100)	100 (100–100)	100 (16.7–100)	100 (50.0–100)
Energy/Fatigue	50.0 (23.8–73.8)	60.0 (37.5–75.0)	55.0 (50.0–80.0)	45.0 (27.5–62.5)	52.5 (38.8–55.5)
Emotional well-being	70.0 (67.0–88.0)	72.0 (68.0–88.0)	84.0 (76.0–88.0)	80.0 (58.0–82.0)	72.0 (53.0–90.0)
Social functioning	68.8 (46.9–100)	100 (65.6–100)	100 (81.3–100)	62.5 (50.0–93.8)	81.3 (62.5–96.9)
Pain	78.8 (65.0–90.0)	90.0 (78.1–97.5)	90.0 (73.8–95.0)	90.0 (83.8–100)	95.0 (71.9–100)
General health	65.0 (45.0–72.5)	62.5 (55.0–83.8)	75.0 (50.0–85.0)	65.0 (40.0–77.5)	62.5 (55.0–68.8)
sVAS, median (P25–P75)	4.3 (3.0–5.3)	5.0 (4.0–6.4)	6.0 (4.5–6.0)	4.0 (2.0–5.5)	4.0 (3.5–5.0)
SNOT-22, median (P25–P75)	25.0 (14.3–30.0)	13.0 (9.3–32.5)	11.0 (6.0–15.0)	12.0 (10.0–30.5)	17.5 (15.3–27.8)
NOSE, median (P25–P75)	25.0 (12.5–45.0)	17.5 (7.5–34)	10.0 (10.0–15.0)	20.0 (10.0–52.5)	27.5 (6.5–44)

Abbreviations: MCA1, first minimal cross-sectional area; NOSE, Nasal Obstruction and Septoplasty Effectiveness Scale; PNIF, peak nasal inspiratory flow; PROMs: patient-reported outcome measures; SF-36, 36-item Short Form Survey; Short-QODNS, short version of Questionnaire of Olfactory Disorders - Negative Statements; SNOT-22, 22-item SinoNasal Outcome Test; sVAS, Visual Analogue Scale for sense of smell; TDI, Threshold + Discrimination + Identification.

**Table 4:** Differences in medians and statistical significance ( *p* -values in brackets)

	Within group comparisons				Between groups comparisons a		
	*Treatment group*			*Control group*			
	T _0_ –T _1_	T _1_ –T _2_	T _0_ –T _2_	T _0_ –T _2_	T _0_	T _2_	|ΔT _0_ –T _2_ |
**Sniffin' Sticks**							
TDI	+4.5 (0.15)	+3.5 (0.22)	+8.0 ( **0.005** )**	−0.1 (0.39)	+0.3 (0.57)	+8.4 (0.19)	8.1 (0.06)
Threshold	+2.8 (0.08)	+1.2 (0.57)	+4.0 ( **0.01** )**	+0.4 (0.53)	−2.2 (0.11)	+1.4 (0.33)	3.6 (0.05)*
Discrimination	+1.5 (0.46)	+0.5 (0.28)	+2.0 ( **0.05** )*	+1.5 (0.51)	+1.0 (0.07)	+1.5 (0.46)	0.5 (0.74)
Identification	+1.5 (0.27)	+1.5 (0.38)	+3.0 ( **0.04** )*	+1.0 (0.23)	0.0 (0.32)	+2.0 (0.08)	2.0 (0.07)
**Nasal measurements**							
PNIF, L/min							
Bilateral PNIF	+22.5 (0.06)	+22.5 (0.84)	+45.0 ( **0.04** )*	−	−20.0 (0.20)	−	−
Right PNIF	+20.0 (0.11)	+27.5 (0.77)	+47.5 (0.07)		−20.0 (0.13)		
Left PNIF	+30.0 ( **0.03** )*	−25.0 (0.27)	+5.0 (0.40)		−40.0 (0.06)		
Acoustic rhinometry							
Right MCA1, cm ^2^	0.0 (0.62)	+0.1 (0.29)	+0.1 (0.34)	−	−0.1 (0.45)	−	−
Right nasal volume (0–5), cm ^3^	+2.4 (0.07)	−0.2 (0.66)	+2.2 ( **0.03** )*		−1.2 (0.1)		
Left MCA1, cm ^2^	0.0 (0.72)	−0.1 (0.96)	−0.1 (0.84)		−0.1 (0.38)		
Left nasal volume (0–5), cm ^3^	+2.9 (0.09)	−1.5 (0.90)	+1.4 (0.11)		−2.4 (0.19)		
**PROMs**							
SF-36, %							
Physical functioning	+5.0 (0.55)	−5.0 (0.83)	0.0 (0.74)	0.0 (0.78)	−5.0 (0.63)	−5.0 (0.63)	0.0 (0.56)
Role limitations due to physical health	+10.0 (0.68)	0.0 (0.97)	+10.0 (0.61)	0.0 (0.74)	−37.5 (0.14)	−25.0 (0.14)	10 (0.32)
Role limitations due to emotional problems	0.0 (0.85)	0.0 (0.40)	0.0 (0.47)	0.0 (0.64)	0.0 (0.55)	0.0 (0.56)	0.0 (0.33)
Energy/Fatigue	+10.0 (0.69)	−5.0 (0.62)	+5.0 (0.41)	+7.5 (0.59)	+5.0 (0.77)	+2.5 (0.43)	2.5 (0.95)
Emotional well-being	+2.0 (0.74)	+12.0 (0.44)	+14.0 (0.43)	−8.0 (1.00)	−10.0 (0.67)	+12.0 (0.29)	22 (0.72)
Social functioning	+31.2 (0.24)	0.0 (0.93)	+31.2 (0.31)	+18.8 (0.73)	+6.3 (0.79)	+18.7 (0.37)	12.4 (0.85)
Pain	+11.2 (0.57)	0.0 (0.65)	+11.2 (0.74)	+5.0 (0.96)	−11.2 (0.28)	−5.0 (0.42)	6.2 (0.27)
General health	−2.5 (0.74)	+12.5 (0.97)	+10.0 (0.50)	−2.5 (1.00)	0.0 (1)	+12.5 (0.32)	12.5 (0.72)
sVAS	+0.7 (0.34)	+1.0 (0.84)	+1.7 (0.17)	0.0 (0.69)	+0.3 (0.78)	+2.0 (0.25)	1.7 (0.82)
SNOT-22	−12.0 (0.27)	−2.0 (0.35)	−14.0 ( **0.03** )*	+5.5 (0.53)	+13.0 (0.53)	−6.5 (0.13)	19.5 (0.41)
NOSE	−7.5 (0.48)	−7.5 (0.20)	−15.0 ( **0.05** )*	+7.5 (0.84)	+5.0 (0.93)	−17.5 (0.20)	22.5 (0.31)

Abbreviations: MCA1, first minimal cross-sectional area; NOSE, Nasal Obstruction and Septoplasty Effectiveness Scale; PNIF, peak nasal inspiratory flow; PROMs, patient-reported outcome measures; SF-36, 36-item Short Form Survey; Short-QODNS, short version of Questionnaire of Olfactory Disorders - Negative Statements; SNOT-22, 22-item SinoNasal Outcome Test; sVAS, Visual Analogue Scale for sense of smell; TDI, Threshold + Discrimination + Identification.

Notes: The sign “ + ” indicates an improvement while the sign “ − ” indicates a worsening in the median values. Please note that for the intergroup differences the direction signs have not been used.

Significant *p* -values in bold. Levels of significance: * *p* ≤ 0.05, ** *p* ≤ 0.01.

a Comparison made with reference to treatment group (i.e., Treatment group – Control group).

### Within and Between Groups Comparisons at Follow-ups

**Fig. 1:**
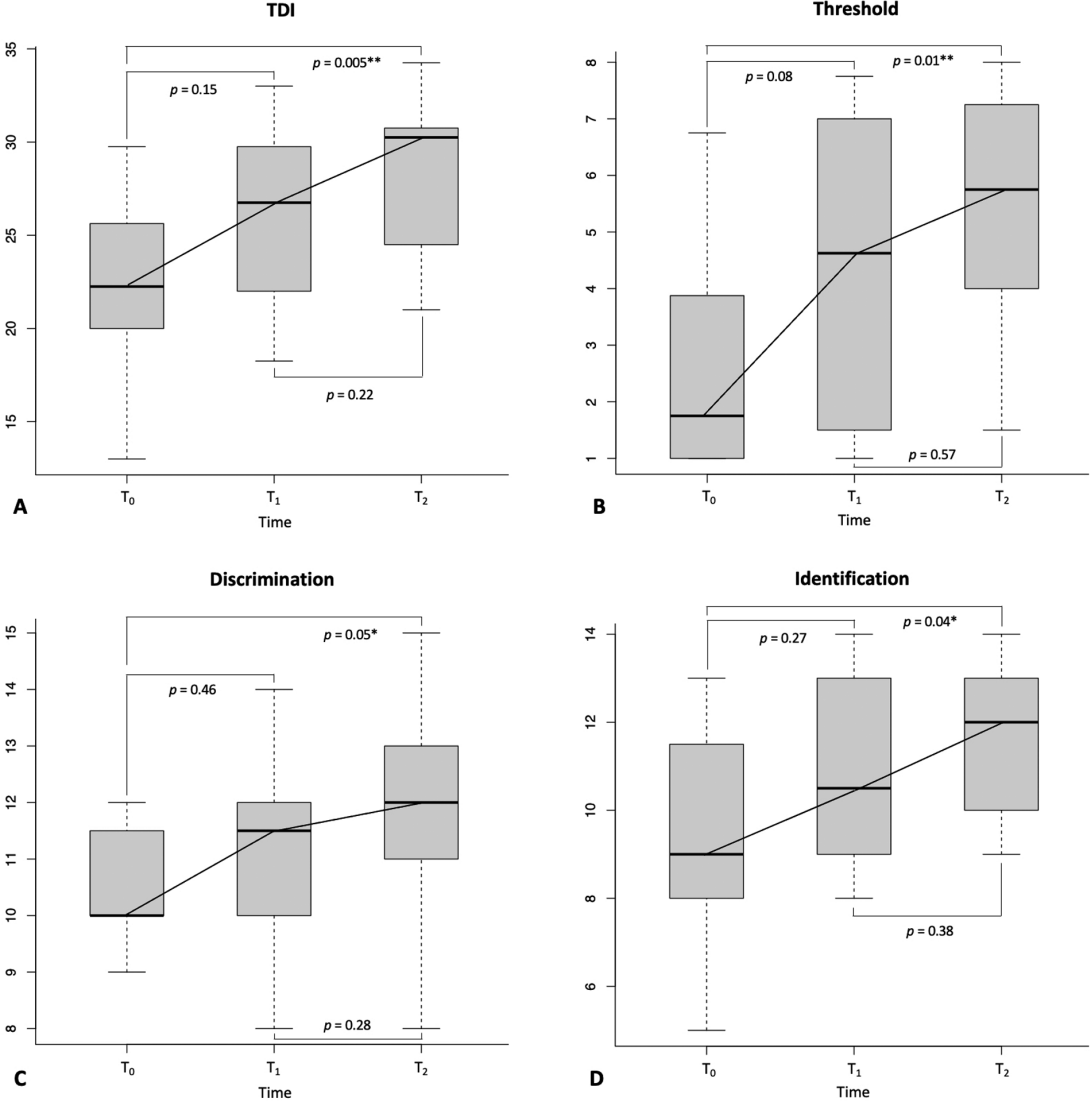
Box plots showing changes in TDI ( **A** ), threshold ( **B** ), discrimination ( **C** ), and identification ( **D** ) scores for the functional septorhinoplasty (fSRP) group during the study period. Statistical difference between intervals is also shown. Levels of significance: * *p* ≤ 0.05, ** *p* ≤ 0.01. TDI, Threshold + Discrimination + Identification.

**Fig. 2:**
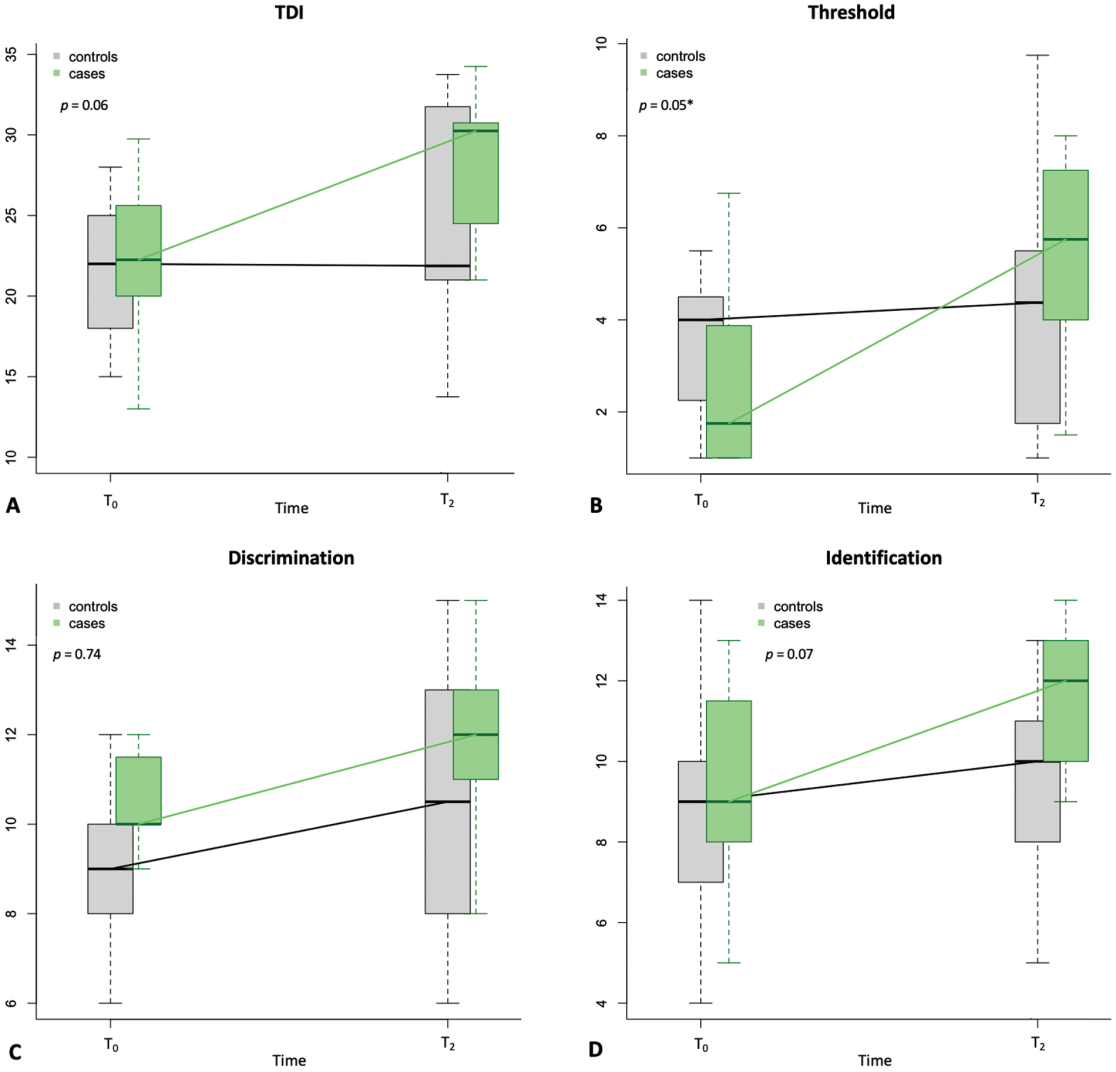
Box plots showing changes in TDI ( **A** ), threshold ( **B** ), discrimination ( **C** ), and identification ( **D** ) scores between the treatment (cases) and the control groups between T _0_ (baseline) and T _2_ (6 months). Statistical difference between intervals is also shown. Levels of significance: * *p* ≤ 0.05. TDI, Threshold + Discrimination + Identification.

An improvement in all S'S scores was observed only in the fSRP group both at T _1_ and T _2_ but these were statistically significant and all above MCID level (apart from discrimination) only at T _2_ ( Fig. 1 *;* Tables 3 and 4 ). A statistically significant improvement at T _2_ from baseline (T _0_ –T _2_ ) was noted only in the treatment group in the bilateral PNIF ( *p* = 0.04) and right NV ( *p* = 0.03), while left PNIF improved significantly only at T _1_ from baseline (T _0_ –T _1_ , *p* = 0.03) ( Tables 3 and 4 ). A statistically significant reduction in the SNOT-22 and NOSE was demonstrated at T _2_ (respectively *p* = 0.03 and *p* = 0.05) only in the treatment group ( Tables 3 and 4 ). When comparing the gain obtained between T _0_ and T _2_ between the two groups, a statistically significant difference was noted for the threshold ( *p* = 0.05) and a trend toward significance was noted for the TDI ( *p* = 0.06) and the identification ( *p* = 0.07), all in favour of fSRP ( Fig. 2 ; Table 4 ).

### Correlation Between Olfactory Function and Nasal Measurements

**Fig. 3:**
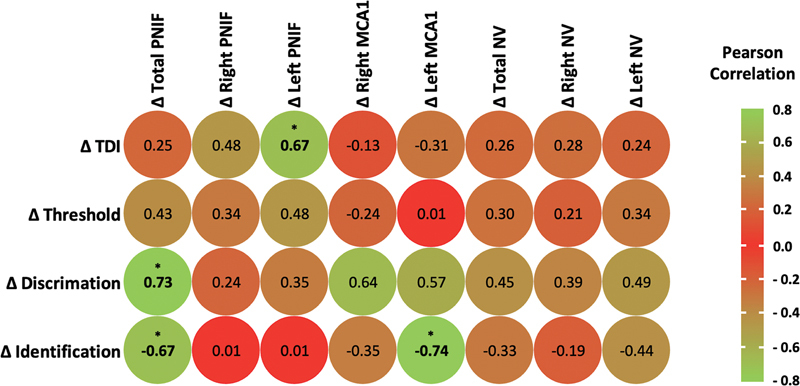
Correlation matrix showing strength of correlations between changes (Δ) in Sniffin' Sticks scores and changes (Δ) in nasal airways parameters in the treatment group. Significant *p* -values in bold. Levels of significance: * *p* ≤ 0.05. MCA1, first minimal cross-sectional area; NV, nasal volume; PNIF, peak nasal inspiratory flow; TDI, Threshold + Discrimination + Identification.

No correlations were found between S'S scores and nasal measurements when considering all the measurements obtained in the whole population. When we looked at the correlations between the changes in S'S scores and nasal measurements between T _0_ and T _2_ in the fSRP group, we found strong significant correlations between changes in left PNIF and changes in TDI ( *r* = 0.67; *p* = 0.05), between changes in total PNIF and changes in discrimination ( *r* = 0.73; *p* = 0.03) and identification ( *r* = − 0.67; *p* = 0.05), and between changes in left MCA1 and changes in identification ( *r* = − 0.74; *p* = 0.03) ( Fig. 3 ).

## Discussion

Our pilot study shows that fSRP can significantly improve persistent C19OD in patients who have previously failed other treatment options for post-infectious OD (PIOD). Patients undergoing fSRP demonstrated a statistically significant improvement in their olfactory scores at 6 months, above MCID level for all S'S scores (apart from discrimination), 33 while an olfactory improvement was not observed in the control arm (patients on OT). Importantly, at 6 months, we observed a statistically significant improvement in threshold gain following fSRP (+3.6 points, *p* = 0.05). A clear positive trend in TDI gain was also observed but did not reach statistical significance (+8.1 points, *p* = 0.06) ( Table 4 ). These olfactory gains represent the olfactory improvement secondary to the intervention minus the control arm, that is, the olfactory benefit obtainable with fSRP when compared to OT. Several studies confirm that olfactory threshold reflects the peripheral olfactory apparatus function (i.e., OE). 10 44 45 This suggests that olfactory benefits following fSRP are primarily related to an increased peripheral olfactory stimulation (i.e., olfactory sensorineural reactivation or sensorineural reversibility) implying that ORNs are still present in patients with persistent C19OD. Our data corroborates previous findings by Whitcroft et al 10 who showed a statistically significant improvement in the mean TDI (+6.5 points, *p* = 0.03) in patients with long-term OD undergoing fSRP. Similarly, we demonstrated TDI improvement in all our fSRP patients, with six of them (66.7%) reaching MCID, 33 when compared to only four (40%) in the control group. Importantly, in the control arm olfaction further decreased in four patients (40%) while deteroriation was not demonstrated in any of the fSRP patients. Although fSRP patients demonstrated a noticeable improvement in their TDI, statistical significance at T _2_ was not reported with patient-reported olfaction (sVAS) and general QoL scores. However, in previous studies we found statistically significant correlations between olfactory scores, sVAS and SF-36, 3 4 and we believe these non-significant improvements obtained in the present study may be related to the small sample size of our cohorts.

The main driving mechanism in the olfactory improvement obtained in the fSRP group is centered around an increase in nasal airflow as confirmed by a strong significant correlation between postoperative changes in S'S scores and PNIF/AR ( Fig. 3 ). Following fSRP, patients experienced an objective and subjective increase in the nasal airflow as demonstrated by a significant improvement of bilateral PNIF ( *p* = 0.04) and a decrease in NOSE and SNOT-22 ( *p* = 0.05 and *p* = 0.03, respectively) at 6 months ( Table 4 ). In addition to this prevalent mechanism, a previous functional MRI study showed that fSRP can lead to structural and functional plasticity of secondary olfactory cortices, caused by a bottom-up plasticity process. 10 In support of this, we demonstrated a statistically significant improvement of the identification and discrimination scores, which have been shown to reflect more complex processing of olfactory information and influenced by cognitive processes. 10 44 45

The concept of olfactory improvement following nasal surgery is not new. 9 15 16 17 18 19 20 21 22 23 24 25 26 However, its efficacy in PIOD, and in particular in C19OD, remains unexplored. Consequentely, in the post-COVID-19 era, in which thousands of people have been left with a debilitating OD unable to improve on other available options, the potential role of fSRP in improving OD is gaining increasing attention from many rhinology surgeons. This notion is supported by recent systematic reviews and meta-analyses showing that fSRP not only constitutes a safe procedure in terms of long-term olfactory function but can also restore smell. 9 30 The majority of studies seem to suggest that an improvement in the nasal airflow in the olfactory area can lead to improved olfaction by enhancing transport of odor molecules to the olfactory cleft. 18 19 23 24 46 47 48 49 50 In particular, a growing body of evidence seems to support the critical role of the INV in influencing airflow in the olfactory cleft region. 28 51 52 53 Spreader grafts are known to increase the INV angle section 54 and, in fact, a positive association between presence of spreader grafts and olfactory outcomes has been reported. 21 Anatomical variation of the ENV can also influence direction of the airflow and play a role in the transportation of odorants to the olfactory cleft. 55 56 Our patients underwent bilateral INV augmentation, by using bilateral spreader grafts, and anatomical variation of the ENV, by means of columellar strut. By increasing the nasal airflow to the olfactory clefts, growing evidence suggests that this increased olfactory stimulation, caused by a greater quantity of odorants reaching the olfactory area, can lead to an improved OE activity. This may contribute to the restoration of the sensorineural deficit (i.e., OE damage) present in C19OD. 10 This increased peripheral input can then lead to a structural and functional plasticity of secondary olfactory cortices through a bottom-up plasticity process. 10

Although nasal airflow improved following fSRP, all patients were enrolled from our long-COVID smell clinic with a primary diagnosis of persistent C19OD and not nasal blockage. However, all patients had a mild nasal blockage with average NOSE scores less than 25 with mildly reduced PNIF/AR scores. Nevertheless, it is important to delineate the separate preoperative diagnoses of C19OD from long-standing mild nasal blockage which appear unrelated in causation prior to COVID. All our patients had reported normal sense of smell prior to COVID-19 (history of OD was an exclusion criteria to the study).

Despite continuous research efforts, treatments for long-term (>1 year) C19OD today remain limited and equally have failed to demonstrate a clinically important olfactory improvement (i.e., above MCID). OT is considered the gold standard treatment for C19OD. 5 57 58 However, its benefits can abate when OD becomes long-standing and our study, unfortunately, seems to suggest so. This paints a bleak picture for those untreated patients with persistent C19OD.

### Strengths and Limitations

To the best of our knowledge, this is the first study to have investigated the role of fSRP, and more widely of nasal airways surgery, in improving sense of smell in patients with C19OD who have failed previous conservative options for PIOD. Moreover, it is the only study that looked into new potential treatments to improve olfaction in patients with a C19OD longer than 2 years, while demonstrating significant olfactory improvement above MCID. The main limitation of our study is the small sample size and, although our study was powered enough at baseline and at 3 months, it losts power at the 6-month follow-up due to patients' dropout.

## Conclusion

Our pilot study suggests that fSRP can significantly improve sense of smell in patients with persistent C19OD lasting more than 2 years with additional significant olfactory threshold gain when compared to OT. By augmenting the INV angle and optimizing nasal airflow to the olfactory cleft, fSRP can improve olfaction by increasing transport of odorants to the OE. This increased stimulation of the olfactory mucosa leads to a sensorineural improvement of the OE potentially triggering a bottom-up plasticity process in the central olfactory areas. Nevertheless, further studies on larger populations are needed to confirm our preliminary findings.
